# Visualization of small visceral arteries on abdominal CT angiography using ultra-high-resolution CT scanner

**DOI:** 10.1007/s11604-021-01124-6

**Published:** 2021-05-04

**Authors:** Kazuya Ogawa, Hiromitsu Onishi, Masatoshi Hori, Atsushi Nakamoto, Takashi Ota, Hideyuki Fukui, Mitsuaki Tatsumi, Yukihiro Enchi, Kazuhiko Sato, Koki Kaketaka, Noriyuki Tomiyama

**Affiliations:** 1 Department of Diagnostic and Interventional Radiology, Osaka University Graduate School of Medicine, 2-2 Yamadaoka, Suita, Osaka 565-0871 Japan; 2 Division of Radiology, Department of Medical Technology, Osaka University Hospital, Suita, Japan

**Keywords:** Image quality, Abdominal CT angiography, High-spatial-resolution CT scanner, Reconstruction technique, Spatial resolution

## Abstract

**Purpose:**

To evaluate the image quality and ability to delineate the small visceral arteries of high-resolution (HR) abdominal CT angiography (CTA) using an ultra-high-resolution computed tomography (UHR CT) scanner.

**Materials and methods:**

Thirty-seven patients were enrolled who underwent abdominal CTA using a UHR CT scanner. The images were reconstructed with a matrix of 1024 × 1024 and 0.25 mm thickness for HR CTA and with a matrix of 512 × 512 and 0.5 mm thickness for normal resolution (NR) CTA. Maximum CT value, image quality, and delineation of the small arteries were compared between HR CTA and NR CTA.

**Results:**

HR CTA showed significantly higher maximum CT value, higher image quality, and better delineation of the small arteries than did NR CTA (*P* < .005).

**Conclusion:**

HR CTA using a UHR CT scanner showed higher image quality than NR CTA and enhanced the delineation of visceral arteries.

## Introduction

Abdominal computed tomography angiography (CTA) is a widely used non-invasive modality to assess abdominal vessel anatomy [1]. Anatomical evaluation of abdominal arteries is important in the diagnosis of vascular diseases, staging of malignant tumors, and planning of surgical or interventional procedures [2–9]. The advantages of CTA compared to other vascular visualization methods such as digital subtraction angiography, ultrasonography, and magnetic resonance angiography, are its non-invasiveness, high spatial and temporal resolution, and/or technical reproducibility [1].

An ultra-high-resolution computed tomography (UHR CT) scanner system has been introduced recently in the clinical setting [10, 11]. The UHR CT system differs mechanically from conventional CT systems in the detector element size and focal spot size and allows the acquisition of images with a higher spatial resolution for both in-plane and body axis directions [10, 12, 13]. The higher spatial resolution of this CT scanner may improve the image quality and delineation of upper abdominal arteries with small diameters.

The purpose of our study was to evaluate the image quality and ability to delineate the small arteries of high-resolution (HR) abdominal CTA with a matrix of 1024 × 1024 using a UHR CT scanner.

## Materials and methods

This retrospective study was approved by our institutional review board, and the requirement to obtain informed consent was waived.

### Patients

The study population comprised 37 consecutive adult patients suspected of having hepatopancreatobiliary cancer, who underwent abdominal multiphasic CT for clinical evaluation using a UHR CT scanner (Aquilion Precision; Canon Medical Systems, Otawara, Japan) at our institute between January and May 2018.

Final diagnosis for these 37 patients [22 males, 15 females; mean age, 70.2 ± 10.1 (39–88) years] was: pancreatic cancer, 14; hepatocellular carcinoma, 10; intraductal papillary mucinous carcinoma, 4; no abnormalities, 4; and one patient each with gallbladder cancer, cholangiocarcinoma, malignant lymphoma, benign pancreatic duct dilation, and benign bile duct dilation.

### CT examination

CT images were acquired using the super-high resolution (SHR) mode of a UHR CT scanner. The scan parameters were: detector configuration, 0.25 mm × 160; tube voltage, 120 kVp; rotation time, 1.0 s; helical pitch, 129/160; focal spot size, 0.6 × 0.6 mm. Tube current was determined by auto exposure control (AEC) according to the patient’s size. The AEC settings were SD of 18 and minimum and maximum current setting was 100 mA and 310 mA, respectively. A use of small focus size setting is required to achieve a high spatial resolution image, effectively. In the setting, the maximum tube current is restricted to 310 mA. To maintain sufficient radiation dose (*i.e.*, mAs), we use the rotation time of 1.0 s. The scan time was about 8 s.

Each patient was given 350 mgI/mL of nonionic iodine contrast medium (Iomeprol; Eisai Co., Ltd., Tokyo, Japan) at a volume of 600mgI/kg for 26 s via the antecubital vein through a 20- or 22-gauge plastic catheter and using a power injector (Dual Shot GX-7; Nemoto Kyorindo Co., Ltd., Tokyo, Japan).

Among the multiphasic CT scans, we used the arterial phase scan image data in the present study. The images were acquired 8 s after the CT value in a region of interest (ROI) had reached the trigger threshold level (> 100 HU). The ROI was placed in the abdominal aorta at the hepatic hilum level, and the contrast medium was tracked with an automatic bolus-tracking technique (SURE Start, Canon Medical Systems).

### Image reconstruction and processing

For HR CTA, early arterial phase images with a matrix of 1024 × 1024, field of view of 400 mm, and 0.25 mm slice thickness were reconstructed. Using the same raw data, images with a matrix of 512 × 512, field of view of 400 mm, and 0.5 mm slice thickness were also reconstructed for normal resolution (NR) CTA, which were configured to match the conventional CT images. To reduce image noise, a full iterative reconstruction technique (FIRST, Canon Medical Systems) was used for these reconstructions (both HR and NR CTA). The FIRST setting of “body standard” was selected on the basis of the preliminary evaluations of reconstruction modes in a small number of cases. Among mild, standard, and strong of the FIRST body and FIRST body sharp, “FIRST body standard” was the best in terms of the balance of image noise and blur.

Coronal partial maximum intensity projection (MIP) images with a 30 mm thick slab were developed from these image sets on the CT scanner console for evaluation of the visceral arteries. HR CTA MIP images completely matched NR CTA MIP images in position for each patient.

### Radiation dose

Volume CT dose index (CTDI _vol_) and dose-length product (DLP) were obtained from the CT scanner.

### Quantitative evaluation

For quantitative evaluation, one radiologist (*blinded data,* with 4 years of experience in abdominal CT) used the partial MIP images of HR and NR CTA on an image workstation (Ziostation2 version 2.4.3.4; Ziosoft Inc., Tokyo, Japan), which can display images with a matrix of 1024 × 1024, to measure full widths at half maximum (FWHMs), maximum CT values, and contrast-to-noise ratio (CNR) for the intra- and extrahepatic small arteries.

As representatives of small arteries, small hepatic arteries of segment VIII and small mesenteric arteries (marginal arteries or vasa recta) were assessed as intra- and extrahepatic small arteries, respectively. Proper hepatic arteries (PHAs) were also assessed as representatives of middle arteries. The radiologist drew lines perpendicularly across the arteries on the workstation to obtain profile curves. The lines on HR and NR CTA MIP images were carefully drawn in the same location for each patient. Based on the data of the profile curves, the FWHMs and maximum CT values were automatically calculated and displayed on the workstation. The radiologist also drew two round ROIs of approximately 20 mm^2^ in the surrounding area on either side of the arteries, avoiding the other anatomical structures. The contrast was calculated by subtracting the average of the CT values of the two ROIs from the maximum CT value of the artery, while the average of standard deviations of the two ROIs was used as the image noise. The CNR was then calculated by dividing contrast by noise.

### Qualitative evaluation

Two other radiologists (*blinded data*, with 21 and 16 years of experience in abdominal CT, respectively) independently reviewed the partial MIP images of HR and NR CTA on a picture archiving and communication system viewer (RapideyeCore; Canon Medical Systems) that can display CT images with a matrix of 1024 × 1024 and performed blinded side-by-side comparisons between HR and NR CTA series for each patient. The displaying sides of the HR or NR CTA series were randomized and the annotations including imaging parameters were blinded on the viewer. The window settings of the image were adjustable by the observers. Finally, the image sets were scored for image quality and ability to delineate the small visceral arteries by using a five-point scale. The spatial resolution was evaluated mostly in terms of the sharpness (or blur) of contours of the organs or vessels and graded as follows: 1, worse; 2, slightly worse; 3, equivalent; 4, slightly better; and 5, better. The image noise was evaluated in terms of the texture of the image and graded as follows: 1, more severe; 2, slightly more severe; 3, equivalent; 4, slightly lower; and 5, lower. The overall image quality was evaluated in terms of multiple aspects including the subjective spatial resolution, the subjective image noise, and the image artifacts and graded as follows: 1, worse; 2, slightly worse; 3, equivalent; 4, slightly better; and 5, better. The delineation of the arteries was evaluated in terms of the conspicuity of the small artery branches. When the difference was obvious, the image was graded as 1 or 5. When the difference was slight, the image was graded as 2 or 4.

### Statistical analysis

The results for HR and NR CTA were compared by using the paired *t* test and the Wilcoxon signed-rank test for the quantitative and the qualitative evaluations, respectively. A two-tailed *P* value of < 0.05 was considered statistically significant.

Inter-reader agreement was computed by using a square-weighted kappa coefficient (weights of 1, 0.94, 0.75, 0.44, and 0 for differences of 0, 1, 2, 3, and 4 categories, respectively) for the four-point ordinal measures [14]. Kappa values of up to 0.40 were considered to indicate positive but poor agreement; 0.41–0.75, good agreement; and 0.75 or higher, excellent agreement [15].

## Results

### Radiation dose

The mean CTDI _vol_ and mean DLP were 15.4 ± 3.5 mGy and 469.5 ± 134.5 mGy · cm, respectively.

### Quantitative evaluation

**Fig. 1:**
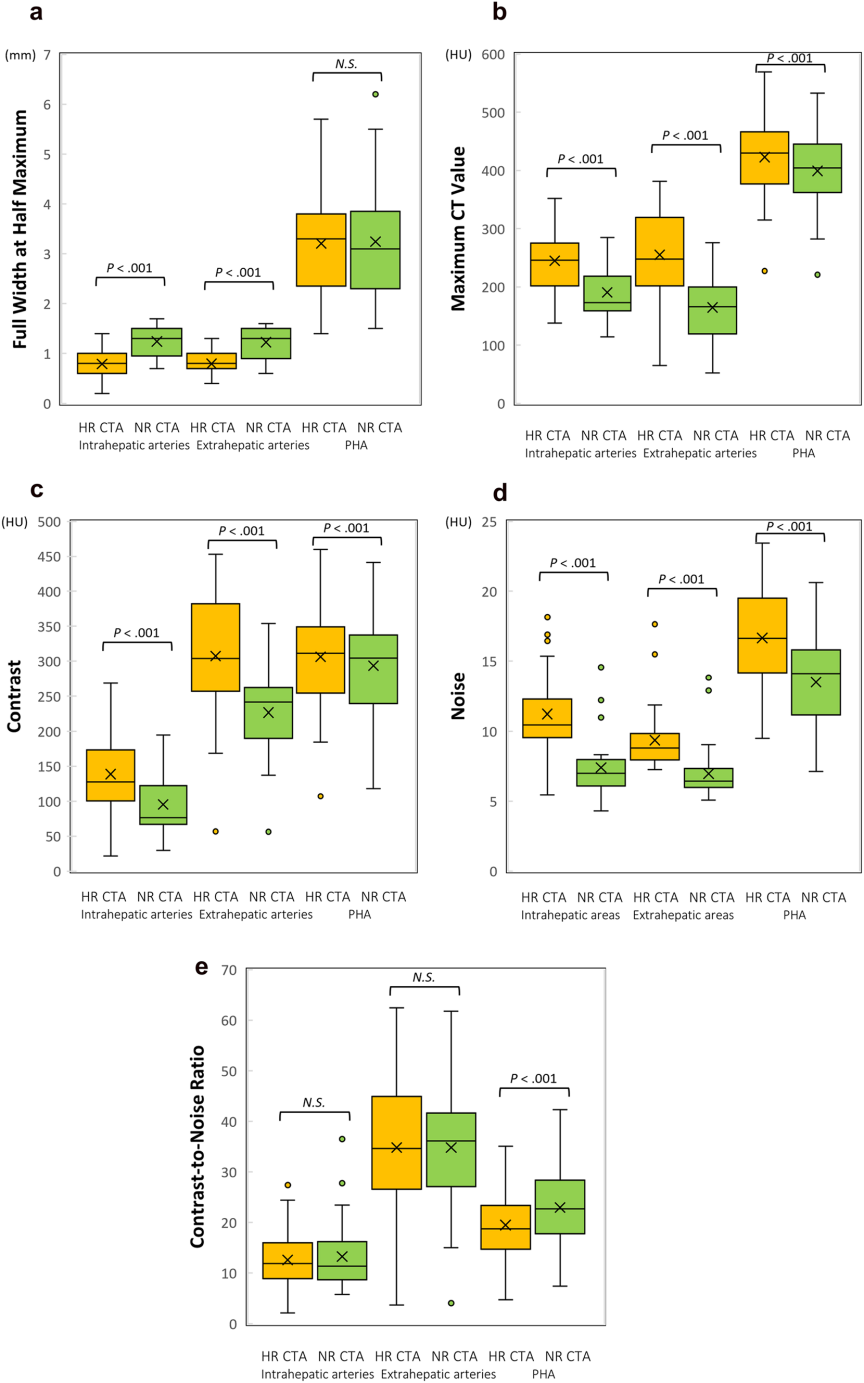
Graphs show (**a**): FWHMs of the arteries, **b**: maximum CT values of the arteries, **c**: contrast between the arteries and the surrounding tissues, **d**: image noise, and **e**: CNR between the arteries and surrounding tissues. All these numbers were measured from the coronal image of the partial MIP. FWHMs of intra- and extrahepatic arteries were significantly less for HR CTA than for NR CTA. FWHMs of PHAs showed no significant differences between HR CTA and NR CTA. Maximum CT values of intra-, extrahepatic arteries, and PHAs were significantly higher for HR CTA than for NR CTA. Contrast between intra-, extrahepatic arteries, and PHAs and surrounding tissues was significantly higher for HR CTA than for NR CTA, while image noise at the intra-, extrahepatic areas, and areas around PHAs was significantly higher for HR CTA than for NR CTA. CNR between the intra- and extrahepatic arteries and the surrounding tissues showed no significant differences between HR CTA and NR CTA. CNR between the PHAs and the surrounding tissues showed significantly higher for NR CTA than for HR CTA

FWHMs of the intrahepatic arteries were significantly less for HR CTA (0.79 ± 0.26 mm) than for NR CTA (1.24 ± 0.30 mm, *P* < 0.001). FWHMs of the extrahepatic arteries were also significantly less for HR CTA (0.79 ± 0.20 mm) than for NR CTA (1.23 ± 0.28 mm, *P* < 0.001). FWHMs of the PHAs showed no significant differences between HR CTA (3.21 ± 1.03 mm) and NR CTA (3.24 ± 1.14 mm, *P* = 0.54) (Fig. 1a).

Maximum CT values of the intrahepatic arteries were significantly higher for HR CTA (244.8 ± 55.4 HU) than for NR CTA (190.6 ± 44.9 HU, *P* < 0.001). Maximum CT values of the extrahepatic arteries were also significantly higher for HR CTA (255.0 ± 72.4 HU) than for NR CTA (164.5 ± 53.8 HU, *P* < 0.001). Maximum CT values of the PHA were significantly higher for HR CTA (422.6 ± 72.3 HU) than for NR CTA (399.1 ± 65.5 HU, *P* < 0.001) (Fig. 1b).

Contrast between the intrahepatic arteries and the surrounding tissues was significantly higher for HR CTA (138.7 ± 56.8 HU) than for NR CTA (95.3 ± 44.0 HU, *P* < 0.001), and that between the extrahepatic arteries and the surrounding tissues was also significantly higher for HR CTA (307.3 ± 83.4 HU) than for NR CTA (226.5 ± 60.4 HU, *P* < 0.001). Contrast between the PHAs and the surrounding tissues was significantly higher for HR CTA (306.2 ± 72.8 HU) than for NR CTA (293.5 ± 67.1 HU, *P* < 0.001) (Fig. 1c).

Image noise at the intrahepatic areas was significantly higher for HR CTA (11.2 ± 2.5 HU) than for NR CTA (7.4 ± 2.1 HU, *P* < 0.001), and that at the extrahepatic areas was also significantly higher for HR CTA (9.4 ± 2.1 HU) than for NR CTA (7.0 ± 1.8 HU, *P* < 0.001). Image noise at the areas surrounding PHA was significantly higher for HR CTA (16.7 ± 3.8 HU) than for NR CTA (13.5 ± 3.2 HU, *P* < 0.001) (Fig. 1d).

CNR for the intrahepatic arteries and the surrounding tissues showed no significant differences between HR CTA (12.6 ± 5.3) and NR CTA (13.3 ± 6.6, *P* = 0.31), while that for the extrahepatic arteries and the surrounding tissues also yielded no significant differences between HR CTA (34.8 ± 12.4) and NR CTA (34.8 ± 12.2, *P* = 0.99). CNR for the PHAs and the surrounding tissues showed significantly higher for NR CTA (23.0 ± 7.5) than for HR CTA (19.5 ± 6.7, *P* < 0.001) (Fig. 1e).

### Qualitative evaluation

**Fig. 2:**
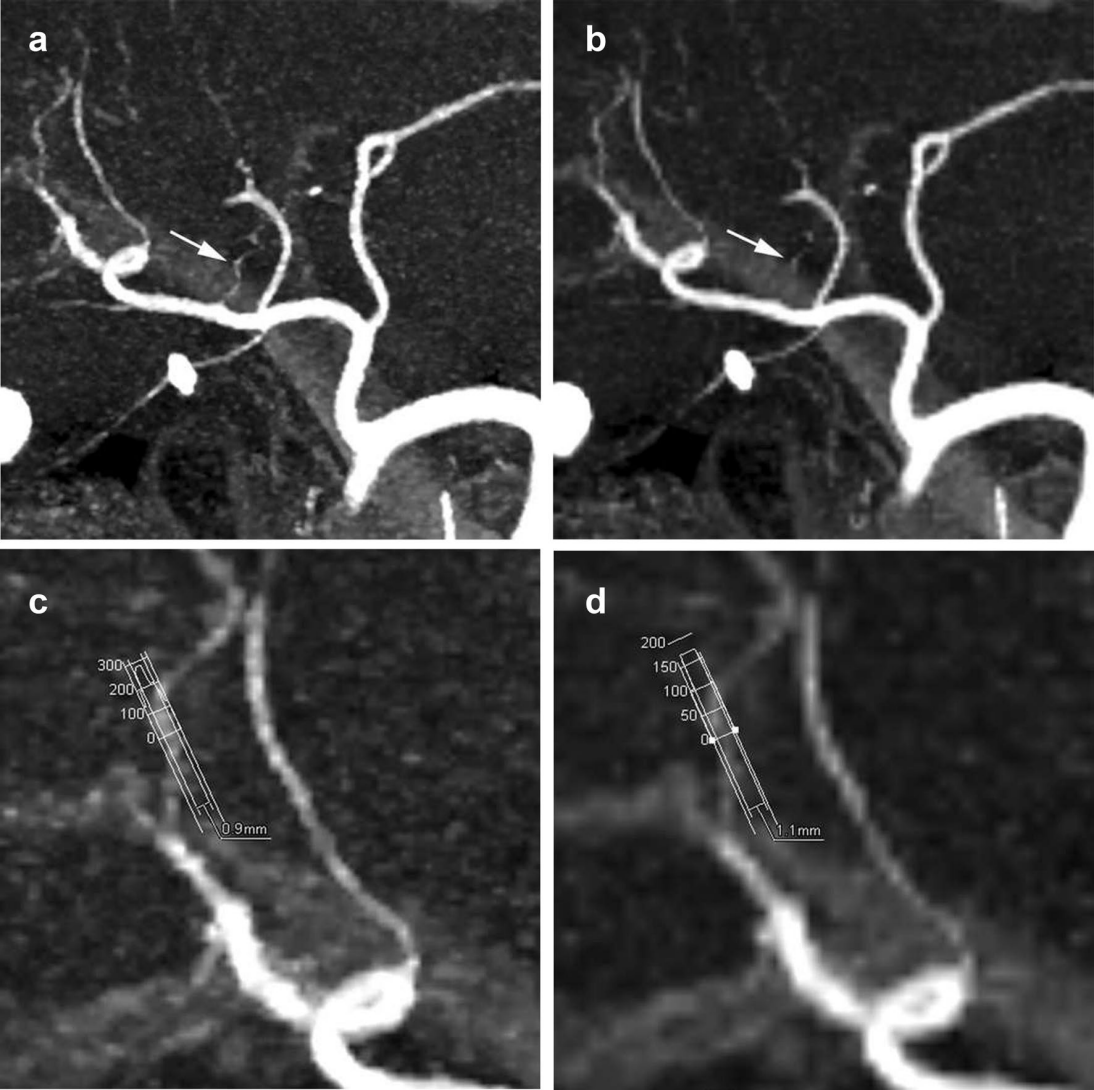
Partial MIP image of hepatic CTA. **a**: HR CTA with a matrix of 1024 × 1024 using the UHR CT scanner more clearly showed a small branch (A1) of the hepatic artery (arrow) than (**b**): NR CTA with a matrix of 512 × 512. Profile curves of the small hepatic artery of segment VIII of (**c**): HR CTA and **d** : NR CTA

**Fig. 3:**
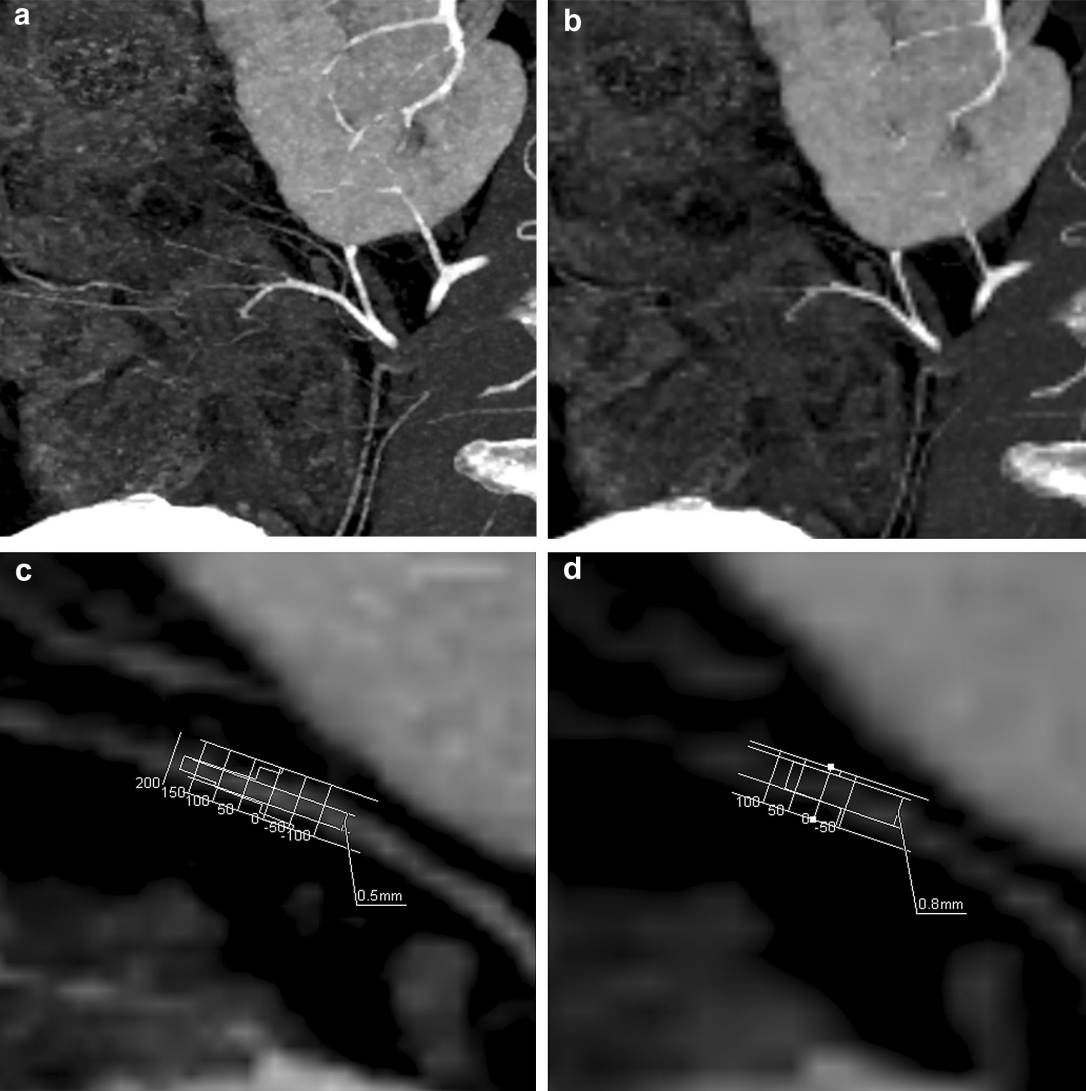
Partial MIP image of CTA of SMA branches. **a** : HR CTA with a matrix of 1024 × 1024 more accurately delineated the marginal arteries and vasa recta than (**b**): NR CTA with a matrix of 512 × 512. Profile curves of the small mesenteric artery of (**c**): HR CTA and **d**: NR CTA

**Fig. 4:**
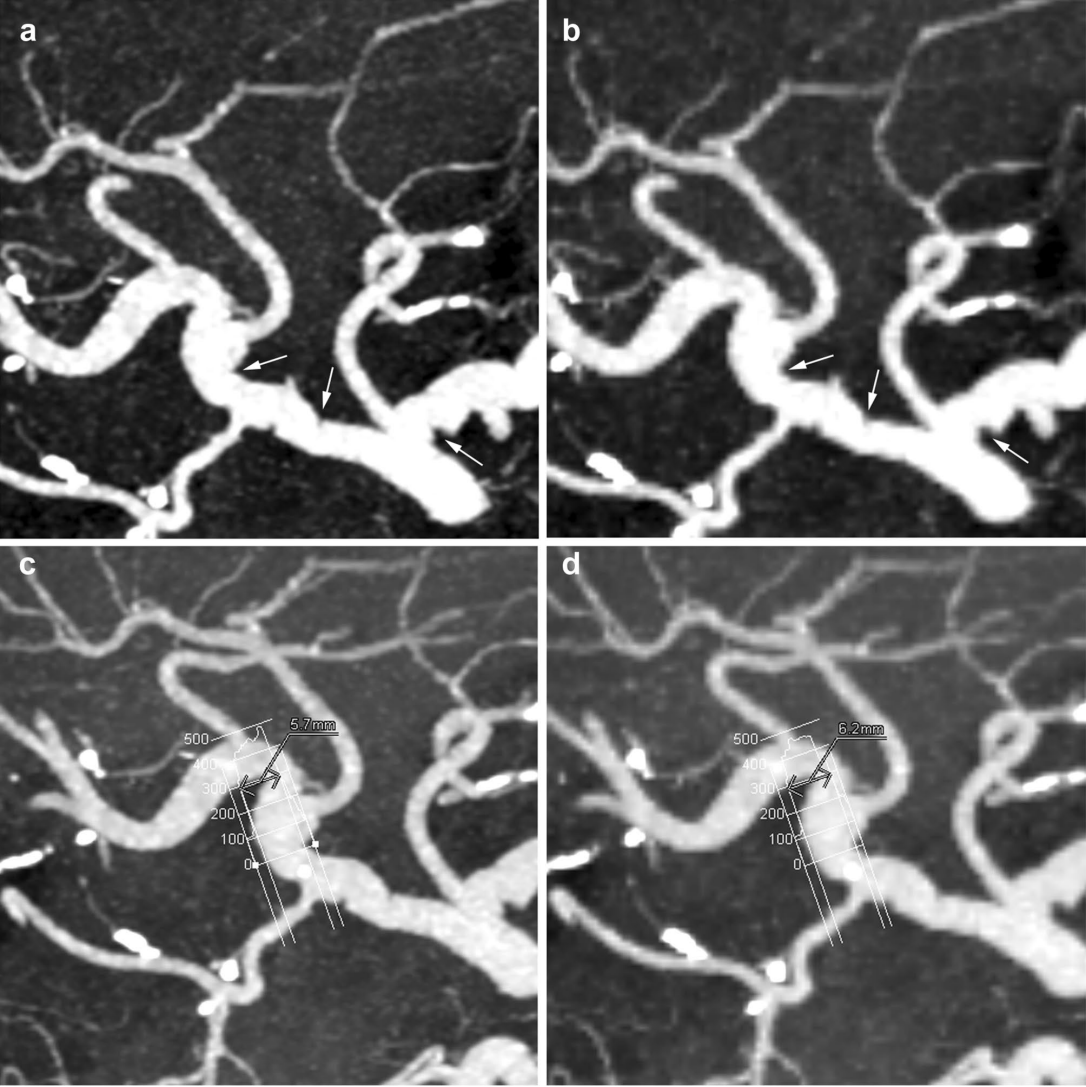
Partial MIP image of CTA of branches of the celiac axis. **a**: HR CTA with a matrix of 1024 × 1024 more clearly showed caliber irregularities of the common hepatic artery, splenic artery, and PHA (arrows) due to the encasement of pancreatic cancer than (**b**): NR CTA with a matrix of 512 × 512. Profile curves of the PHA of (**c**): HR CTA and **d**: NR CTA

HR CTA showed significantly higher spatial resolution, more severe image noise, higher overall image quality, and better delineation of the intra- and extrahepatic arteries than NR CTA (*P* < 0.005, for each feature) (Figs. 2, 3, 4) (Table 1). Inter-reader agreement between the two readers was excellent for evaluation of image quality (kappa: 0.83, 0.84, and 0.77 for sharpness, image noise, and overall image quality, respectively) and excellent for evaluation of delineation of the extrahepatic arteries (kappa: 0.85).

**Table 1:** Results of qualitative evaluation

	Radiologist 1			Radiologist 2		
	Mean Score		*P* value	Mean Score		*P* value
	HR CTA	NR CTA		HR CTA	NR CTA	
Spatial resolution	4.4	1.6	< .001*	3.9	2.1	< .001*
Image noise	1.7	4.3	< .001*	2.1	4.0	< .001*
Overall image quality	4.0	2.0	< .001*	3.7	2.3	< .001*
Delineation of the intrahepatic arteries	3.4	2.6	< .005*	3.4	2.6	< .001*
Delineation of the extrahepatic arteries	4.0	2.0	< .001*	4.0	2.0	< .001*

The results for HR and NR CTA were compared by using the Wilcoxon signed-rank test for the qualitative evaluations

*HR CTA* high resolution CT angiography, *NR CTA* normal resolution CT angiography

*There was a significant difference between HR CTA and NR CTA (*P* < .05)

## Discussion

Our results showed that the overall image quality and the delineation of small visceral arteries for HR CTA were significantly better than that for NR CTA.

Although several studies of CTA using a UHR CT scanner were reported, there were no reports concerning small arteries in the abdomen. In the clinical practice, the evaluations of the visceral arteries are essential in many situations (*e.g.*, mapping of the hepatic arteries for the TACE, mapping of the gastric or mesenteric arteries for the surgical resection of the gastric or colorectal cancers, and evaluation for the resectability of pancreatic cancer). Therefore, the delineation of the small abdominal arteries may be potentially helpful to these clinical situations. On the other hand, the abdominal CTA using a UHR CT scanner may be challenging in the aspect of the restriction of the radiation dose in the use of the small focus size and the increase of image noise. We achieved the abdominal CTA using a UHR CT scanner with good image quality and acceptable radiation dose by means of the optimal scan setting and a use of full iterative reconstruction technique (*i.e.*, FIRST).

HR CTA showed higher maximum CT values for the arteries than did NR CTA, which is considered to be caused by a reduction in the averaging effect of CT values within individual pixels due to the higher spatial resolution. The higher maximum CT values for HR CTA contribute to the higher contrast between the small arteries and the surrounding tissues.

The higher spatial resolution can cause an increase in image noise in CT images [10, 16], so that the noise for HR CTA was higher than that for NR CTA in our results. However, CNR for HR CTA was comparable to that for NR CTA, since the image contrast for HR CTA was higher than that for NR CTA in the analysis of small arteries. As for the middle artery, CNR for NR CTA was significantly higher than that for HR CTA due to the larger increase in image noise compared to that in contrast. The findings of the qualitative study showed that the overall image quality of HR CTA was superior to that of NR CTA despite the higher image noise for HR CTA in the small arteries.

FWHM is often used as an index of spatial resolution. For this purpose, the target object less than one pixel is required to evaluate the spatial resolution of CT images. However, the blood vessels evaluated in the study may be larger than one pixel. So, it is impossible to prove that the spatial resolution is high due to the small FWHM. FWHM is also used to measure the diameter of vessels or other anatomical structures in the clinical study. The FWHM values of the small arteries in the present study were higher in NR CTA than in HR CTA. It has been reported that this method overestimates the diameter for the small vessels with a diameter of 2 mm or less when measured using ordinary spatial resolution CT scanners [17]. The values in NR CTA may have been also overestimated.

In the present study, pixel size of the reconstructed images for HR CTA and NR CTA used were approximately 0.39 mm (field of view, 400 mm; matrix size, 1024) and 0.78 mm (field of view, 400 mm; matrix size, 512), respectively. For UHR CT, the 10% modulation transfer function is approximately 13.0 line pairs per centimeter with the standard kernel [10], which corresponds to a 0.38 mm in-plane spatial resolution. The pixel size of HR CTA images was approximately equal to the radical spatial resolution, which was determined by the mechanical architecture, e.g., detector segmentation and focal spot size, of the UHR CT scanner. A matrix of 1024 × 1024 can thus be of practical use for UHR CT.

A full iterative reconstruction technique significantly reduces noise and improves spatial resolution of abdominal CTA images in a conventional CT scanner compared to results obtained with the filtered back projection method or hybrid-iterative reconstruction technique [18]. The full iterative reconstruction technique may also have substantially reduced image noise and enhanced the spatial resolution for HR CTA using a UHR CT scanner in the present study. The reconstruction time for HR CTA using FIRST was about 25 min, which is considered acceptable for clinical use. The data size of HR CTA was eight times larger than that of NR CTA.

The precise visualization of small visceral arteries has the potential clinical benefits of contributing to the selection of the tumor feeding arteries in less time in the transarterial chemoembolization for the hepatocellular carcinomas, the accurate evaluation of the vascular invasion of the tumors, the reduction of the volume of bleeding, or the preservation of the required arteries in the various surgical procedures.

CTDI _vol_ and DLP were used as indicators of patient radiation exposure. In previous studies concerning upper abdominal CTA using conventional CT scanners, mean CTDI _vol_ was found to be 14.99 or 16.21 mGy and mean DLP 497.6 or 516.33 mGy · cm [19]. The mean CTDI _vol_ and DLP determined in the present study (15.4 mGy and 469.5 mGy · cm, respectively) were considered to be almost comparable.

Our study has several limitations. First, it was a retrospective single-institution study with a small number of subjects. Second, NR CTA, which yields a CTA image with conventional spatial resolution, was reconstructed from the same raw data as HR CTA. Images with a matrix of 512 × 512 and 0.5 mm slice thickness obtained with a UHR CT scanner may differ in image quality from those obtained with a conventional CT scanner.

In conclusion, HR CTA using a UHR CT scanner showed better image quality than NR CTA and improved the delineation of small visceral arteries. It may thus contribute to more accurate evaluation of small arteries.
